# Detection of Spatiotemporal Clusters of COVID-19–Associated Symptoms and Prevention Using a Participatory Surveillance App: Protocol for the @choum Study

**DOI:** 10.2196/30444

**Published:** 2021-10-06

**Authors:** David De Ridder, Andrea Jutta Loizeau, José Luis Sandoval, Frédéric Ehrler, Myriam Perrier, Albert Ritch, Guillemette Violot, Marc Santolini, Bastian Greshake Tzovaras, Silvia Stringhini, Laurent Kaiser, Jean-François Pradeau, Stéphane Joost, Idris Guessous

**Affiliations:** 1 Division of Primary Care Geneva University Hospitals Geneva Switzerland; 2 Department of Health and Community Medicine Faculty of Medicine University of Geneva Geneva Switzerland; 3 Group of Geographic Information Research and Analysis in Population Health Geneva University Hospitals Geneva Switzerland; 4 Laboratory of Geographic Information Systems School of Architecture, Civil and Environmental Engineering Swiss Federal Institute of Technology Lausanne Lausanne Switzerland; 5 Department of Oncology Geneva University Hospitals Geneva Switzerland; 6 Direction of Information Systems Geneva University Hospitals Geneva Switzerland; 7 Communication Directorate Geneva University Hospitals Geneva Switzerland; 8 Center for Research and Interdisciplinarity INSERM U1284 University of Paris Paris France; 9 Division of Infectious Disease and Laboratory of Virology Geneva University Hospitals Geneva Switzerland; 10 Center for Emerging Viral Diseases Geneva University Hospitals Geneva Switzerland

**Keywords:** participatory surveillance, infectious disease, COVID-19, SARS-CoV-2, space-time clustering, digital health, mobile app, mHealth, epidemiology, surveillance, digital surveillance, public health

## Abstract

**Background:**

The early detection of clusters of infectious diseases such as the SARS-CoV-2–related COVID-19 disease can promote timely testing recommendation compliance and help to prevent disease outbreaks. Prior research revealed the potential of COVID-19 participatory syndromic surveillance systems to complement traditional surveillance systems. However, most existing systems did not integrate geographic information at a local scale, which could improve the management of the SARS-CoV-2 pandemic.

**Objective:**

The aim of this study is to detect active and emerging spatiotemporal clusters of COVID-19–associated symptoms, and to examine (a posteriori) the association between the clusters’ characteristics and sociodemographic and environmental determinants.

**Methods:**

This report presents the methodology and development of the @choum (English: “achoo”) study, evaluating an epidemiological digital surveillance tool to detect and prevent clusters of individuals (target sample size, N=5000), aged 18 years or above, with COVID-19–associated symptoms living and/or working in the canton of Geneva, Switzerland. The tool is a 5-minute survey integrated into a free and secure mobile app (CoronApp-HUG). Participants are enrolled through a comprehensive communication campaign conducted throughout the 12-month data collection phase. Participants register to the tool by providing electronic informed consent and nonsensitive information (gender, age, geographically masked addresses). Symptomatic participants can then report COVID-19–associated symptoms at their onset (eg, symptoms type, test date) by tapping on the @choum button. Those who have not yet been tested are offered the possibility to be informed on their cluster status (information returned by daily automated clustering analysis). At each participation step, participants are redirected to the official COVID-19 recommendations websites. Geospatial clustering analyses are performed using the modified space-time density-based spatial clustering of applications with noise (MST-DBSCAN) algorithm.

**Results:**

The study began on September 1, 2020, and will be completed on February 28, 2022. Multiple tests performed at various time points throughout the 5-month preparation phase have helped to improve the tool’s user experience and the accuracy of the clustering analyses. A 1-month pilot study performed among 38 pharmacists working in 7 Geneva-based pharmacies confirmed the proper functioning of the tool. Since the tool’s launch to the entire population of Geneva on February 11, 2021, data are being collected and clusters are being carefully monitored. The primary study outcomes are expected to be published in mid-2022.

**Conclusions:**

The @choum study evaluates an innovative participatory epidemiological digital surveillance tool to detect and prevent clusters of COVID-19–associated symptoms. @choum collects precise geographic information while protecting the user’s privacy by using geomasking methods. By providing an evidence base to inform citizens and local authorities on areas potentially facing a high COVID-19 burden, the tool supports the targeted allocation of public health resources and promotes testing.

**International Registered Report Identifier (IRRID):**

DERR1-10.2196/30444

## Introduction

### Background

Since 2020, many countries worldwide, including Switzerland, have experienced multiple fluctuations of SARS-CoV-2 transmission associated with cycles of increased followed by relaxed measures [1,2]. These cycles will remain until appropriate immunization levels through natural infection or vaccination are achieved [3]. There is growing concern that adherence to public health recommendations, including SARS-CoV-2 testing, is declining (ie, testing fatigue) [4,5]. Thus, it is essential to define alternative strategies to encourage timely testing, recommendation compliance, and monitor spread of the virus.

Traditional disease surveillance systems, relying on SARS-CoV-2 cases confirmed by real-time polymerase chain reaction (RT-PCR) or antigenic tests, suffer from underreporting and a delay of notification due to the time between the onset of symptoms and case declaration [6]. There is a need for alternative sources of information complementing the traditional COVID-19 surveillance systems [7,8]. The use of collaborative information through participatory surveillance, facilitated by mobile phone apps and web-based tools, offers a quick way to collect data at a population scale and disseminate critical information [9]. Participatory syndromic surveillance systems have shown promising results for several public health events [10,11], and many have already been described since the start of the COVID-19 pandemic [9,12-16]. However, most apps require users to report health information daily or weekly, which could increase dropout rates. Moreover, only a few apps have been configured to collect geographic information at high resolution [12,15,16]. Increased knowledge about the potential risk of infection at a local scale (ie, precise geographic coordinates associated with a specific date) can play an essential role for residents and local authorities [17] who lack information to make appropriate decisions [18]. Several studies have emphasized the importance of rapid prospective spatiotemporal epidemiological surveillance, which can help prioritize locations for targeted interventions, rapid testing, and resource allocation [7,19-22]. Despite this opportunity, individuals may be reluctant to report exact addresses together with symptoms owing to security and privacy concerns [23,24]. Therefore, such participatory surveillance systems must be developed in a way that minimizes the risks and best guarantees the individuals’ rights to privacy.

The @choum (English: “achoo”) study proposes a digital participatory epidemiological surveillance tool using precise space-time data to monitor COVID-19–associated symptoms at their onset in the canton of Geneva, Switzerland. The @choum tool is a 5-minute survey that profits from being integrated into a free and secure preexisting app for COVID-19 resources and information developed by the University Hospitals of Geneva (HUG). Upon registration to @choum, participants can report COVID-19–associated symptoms at their onset by tapping on the @choum button visible on the home screen. They are then redirected to the official COVID-19 recommendations websites. This study provides an innovative combination of best practices in participatory surveillance and spatiotemporal epidemiology. This report describes the rationale and methodology of the @choum study, and presents the development procedure of the @choum digital tool, which has been publicly available since February 11, 2021.

### Objectives

The specific study objectives are as follows: (1) to prospectively detect active and emerging spatiotemporal clusters of reported COVID-19–associated symptoms by running a daily automated spatiotemporal cluster detection algorithm; and (2) to retrospectively analyze the outbreaks detected over the 12-month data collection phase, and examine the association of outbreak characteristics with sociodemographic and environmental factors.

## Methods

### Study Design

The @choum study evaluates a digital participatory epidemiological surveillance tool to detect and prevent spatiotemporal clusters of individuals, aged 18 years or above, with COVID-19–associated symptoms living and/or working in the canton of Geneva, Switzerland. Its design integrates both prospective and retrospective analytical features. The study began on September 1, 2020, and will be completed on February 28, 2022.

### Setting and Governance Team

This study takes place in the canton of Geneva. It is carried out by an interdisciplinary team based at the HUG in close collaboration with experts from the University of Geneva (UNIGE), the Swiss Federal Institute of Technology Lausanne (EPFL), and the University of Paris (UP). The different components of the study have been distributed across the governance team members as follows: digital tool development, Direction of Information Systems, HUG; communication campaign strategy, Communication Directorate, HUG; spatiotemporal analyses, Geographic Information Research and Analysis in Population Health, HUG, UNIGE, and EPFL; and smart testing referral strategy, Division of Primary Care, HUG. Collaborators at the Center for Research and Interdisciplinarity at UP provide expertise on citizen-based participation strategies. Prior research has demonstrated our extended team’s productive collaboration, generating impactful research that integrates expertise in the fields of population health, spatial epidemiology, and virology [21,22,25].

### Participants and Eligibility

Participant eligibility criteria include (i) aged≥18 years, (ii) living and/or working in the canton of Geneva, (iii) having access to a smartphone with a Swiss or French phone number and a connection to the internet, and (iv) being capable of providing informed consent. Individuals not meeting these criteria are prevented from completing study registration but are redirected to official recommendation websites. All participants provide electronic informed consent (eIC) to participate in the study. Participant enrollment began on February 11, 2021, and will be completed on February 28, 2022, meaning that participants can enroll during the entire data collection phase.

Our approach to eIC follows the guidelines provided by the local institutional ethics committee, and requires participants to (i) read the study electronic information letter and declaration of consent displayed within two scrolling menus, (ii) check the consent box, and (iii) tap the “I accept” button. The study does not ask for the name or signature of participants to ensure individual privacy. Participants are informed that they can opt out at any time via their profile or by contacting the research team. In the absence of an opt-out request, all participants are followed throughout the data collection phase.

### Study Preparation

A 5-month preparation phase, starting on September 1, 2020, has preceded the 12-month data collection phase. The development milestones included: (1) frontend development (content, design, and branching logic); (2) backend development (database); (3) spatiotemporal clustering analysis implementation and testing; (4) data exchange between the app, server, and Python script running daily clustering analyses; and (5) complete security assessment. Following an iterative process, the tool’s content was reviewed and refined by experts in infectious disease, epidemiological research, citizen science, health communication, and app design. Multiple technical and analytical tests have been performed at various development stages of the tool in preproduction and production modes.

A 1-month pilot phase was performed among 38 pharmacists working in seven pharmacies located in the canton of Geneva before the study launch. The pharmacists were asked to follow expected app utilization scenarios (eg, reporting either a positive or negative test result) and to complete a 10-minute satisfaction online questionnaire.

### Recruitment Campaign

**Figure 1:**
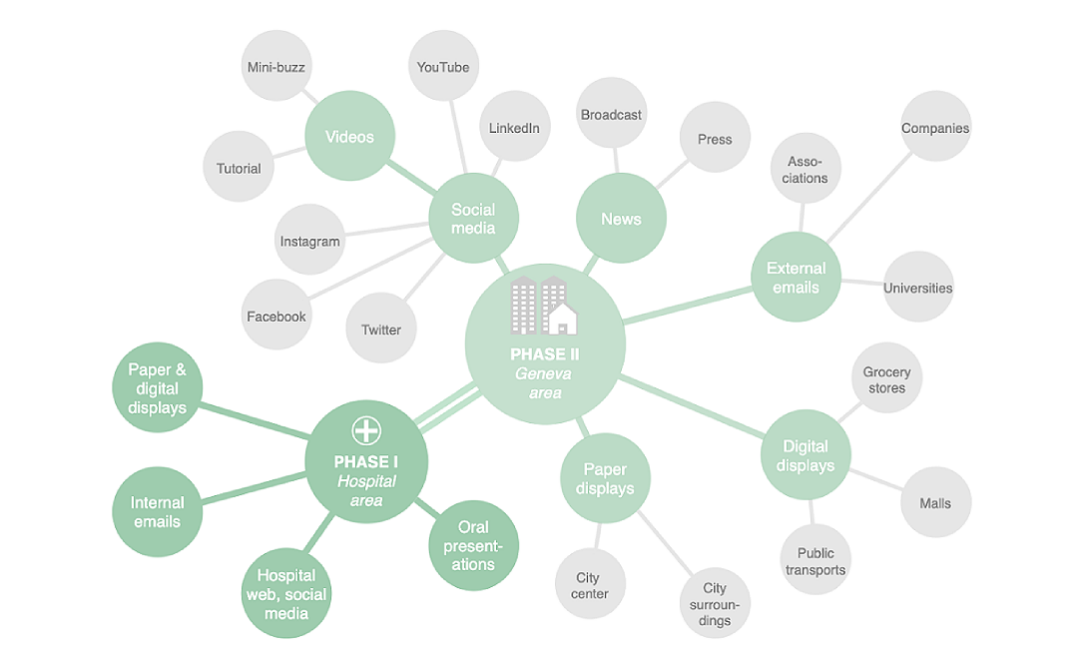
The @choum (English: “achoo”) comprehensive communication campaign started on February 11, 2021, within the hospital setting (Phase I) before its extension to the entire population of the canton of Geneva on March 1, 2021 (Phase II).

We developed a comprehensive two-phase multichannel communication campaign launched together with the @choum tool on February 11, 2021 (Figure 1). This campaign aims to maximize the chances of attaining individuals interested in participating in the study. We conducted a first communication phase of 2 weeks within the hospital (HUG) setting before launching the second phase targeting the entire population of the canton of Geneva. This progressive two-phase communication approach has enabled our research team to maintain control over potential technical challenges associated with an increased participation rate. The first phase of the communication strategy included paper and digital advertisements (flyers, posters, screens), internal emails (intranet), hospital-based web and social media pages (landing page linked to other HUG-based pages, YouTube, LinkedIn, Instagram, Facebook, and Twitter), and oral presentations. The second phase expanded the communication strategy to additionally include outdoor advertisement at locations of high pedestrian traffic in the city center and surroundings areas (main streets, parks, bus and train stations), digital advertisement (public transport, grocery stores, and malls), external emails (intranet of partner associations, local companies, and universities), articles and interviews in traditional media (radio, tv, press), and social media also broadcasting five 2-minute video tutorials and a short and engaging 45-second video [26]. The selection of outdoor advertisement locations was guided by prior research from our group, which identified the canton’s areas at risk of increased SARS-CoV-2 transmission [25]. All communication materials were designed and developed by the HUG communications team in collaboration with the other governance team members. The core advertising message expresses the possibility of contributing to a collective action helping researchers in the fight against the pandemic.

### Participation Steps and Elements

Individuals wishing to participate in the study can download the CoronApp-HUG, freely available on Android (Google Play Store) [27] and iOS (Apple Store) [28] devices in Switzerland and France. @choum has been added as a new functionality to the preexisting CoronApp-HUG mobile app, released by the HUG on March 12, 2020, which provides users with valuable resources and the latest information on COVID-19. Upon download completion, users accept the General Terms and Conditions of Use of the app and access the tool via a button visible on the home screen.

**Figure 2:**
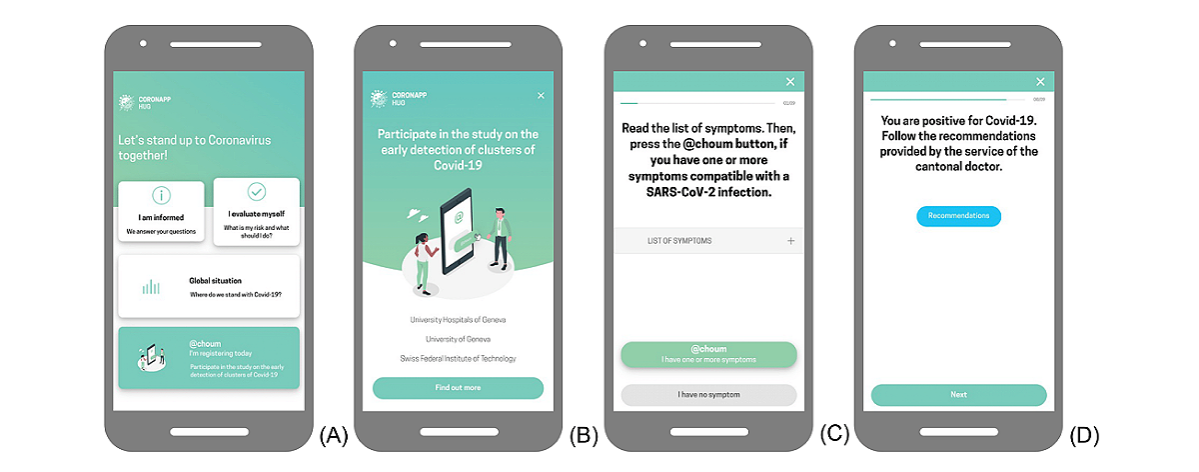
The main @choum (English: “achoo”) study screens, translated into English: (A) CoronApp-HUG home screen with access to the study, (B) the screen preceding study consent and registration, (C) the main screen providing the possibility to report COVID-19–associated symptoms by tapping on the @choum button (green button stating “@choum I have one or more symptoms”), and (D) example screen redirecting to COVID-19 recommendations.

**Figure 3:**
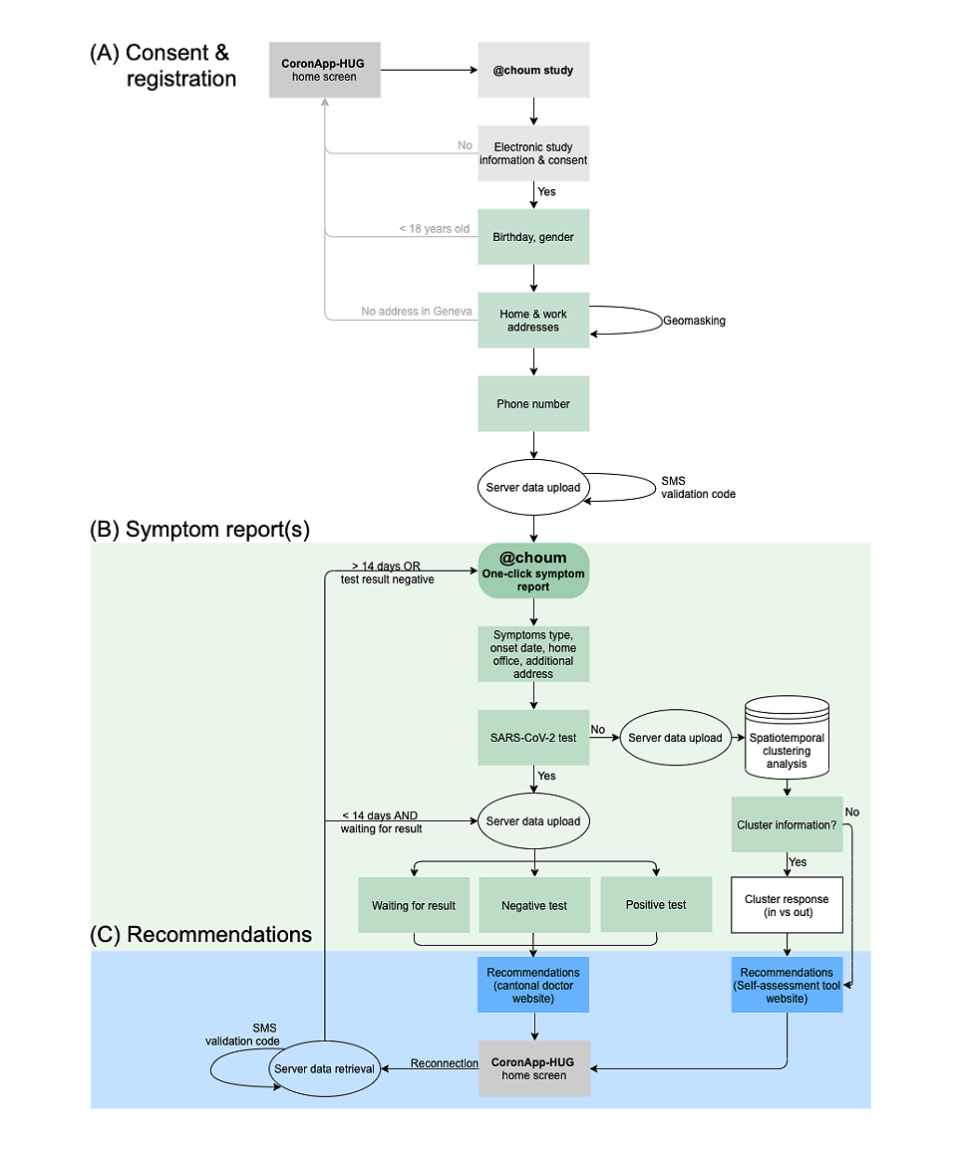
Simplified scheme of the @choum (English: “achoo”) study branching logic and elements. Once (A) registration is completed, (B) COVID-19–associated symptoms can be reported at multiple time points throughout the data collection phase (from February 11, 2021, to February 28, 2022), and (C) recommendations are provided based on each report. HUG: Geneva University Hospitals.

Participation in the @choum study is simple, takes approximately 5 minutes, and consists of three steps: (1) consent and registration, (2) report of COVID-19–associated symptoms by tapping on the @choum button, and (3) COVID-19 recommendations. Registration is completed only once, whereas episodes of COVID-19–associated symptoms can be reported at multiple time points throughout the data collection phase. An information button is displayed on the upper right corner of some selected screens, which provides participants with more detailed explanations relevant to each participation step. The main study screens are presented in Figure 2, and the study branching logic and elements are presented in Figure 3.

First, individuals interested in participating receive information on the study goals, benefits, and impacts of participation. Second, they go through the electronic letter of information and the declaration of consent to give their eIC via the “I accept” button, designed for this purpose. Individuals are informed that they will be asked for their work and home addresses but that these addresses are not used for tracking purposes and are replaced with masked geographic coordinates. The geographic masking procedure consists of displacing geographic coordinates randomly within a circular radius of 200 meters around their original position [29]. Individuals providing eIC can register for the study by reporting their gender, age, residential and/or work addresses, and phone number. Registration is then confirmed by a unique code sent via SMS text message. Upon completion of study registration, the @choum button for symptoms reporting becomes accessible on the home screen. No action is required from participants, as long as they are not experiencing any COVID-19–associated symptoms. A user profile is created, allowing participants to check and modify their home and work addresses at any given time.

When perceiving COVID-19–associated symptoms, participants can start the symptoms report process by tapping on the @choum button. Users are required to go through a list of symptoms before proceeding further into their symptom report. This step aims to ensure that participants are informed about the symptoms typically associated with COVID-19. Participants are asked a set of questions (detailed in the Data Collection section) concerning their symptoms. If they have not yet been tested for SARS-CoV-2, they are given the possibility to be informed on whether they currently live or work in an area where a high number of other participants have reported COVID-19–associated symptoms (ie, active and emerging spatiotemporal clusters). This feedback is based on the automated spatiotemporal clustering analysis results updated daily and returned to participants via specific screens within the app to encourage user engagement. Symptomatic participants with addresses located within such a cluster are further encouraged to get tested for SARS-CoV-2 and subsequently report the test date and result. This information is used to filter the addresses included in the prospective spatiotemporal cluster detection analyses.

Lastly, participants are redirected to publicly available and official recommendation websites based on their reported information. Participants who have symptoms but have not yet been tested for SARS-CoV-2 are redirected to a survey tool by the Swiss Federal Office of Public Health (SFOPH) [30]. The tool allows individuals to self-assess their risk of SARS-CoV-2 infection and book a test appointment through the HUG website. Participants are presented with a link to the cantonal doctor’s websites at each participation step and are invited to follow the official COVID-19 recommendations [31]. Upon symptoms reporting, all participants are thanked for their participation and invited to download the contact-tracing app SwissCovid [32], launched by the SFOPH, which helps interrupt chains of transmission using digital contact tracing of confirmed COVID-19 cases. After a period based on the information provided in the symptom report (Figure 3), the @choum button becomes accessible again and participants have the opportunity to create another report upon experiencing new COVID-19–associated symptoms. All study screens are presented in Multimedia Appendix 1.

### Data Collection

**Figure 4:**
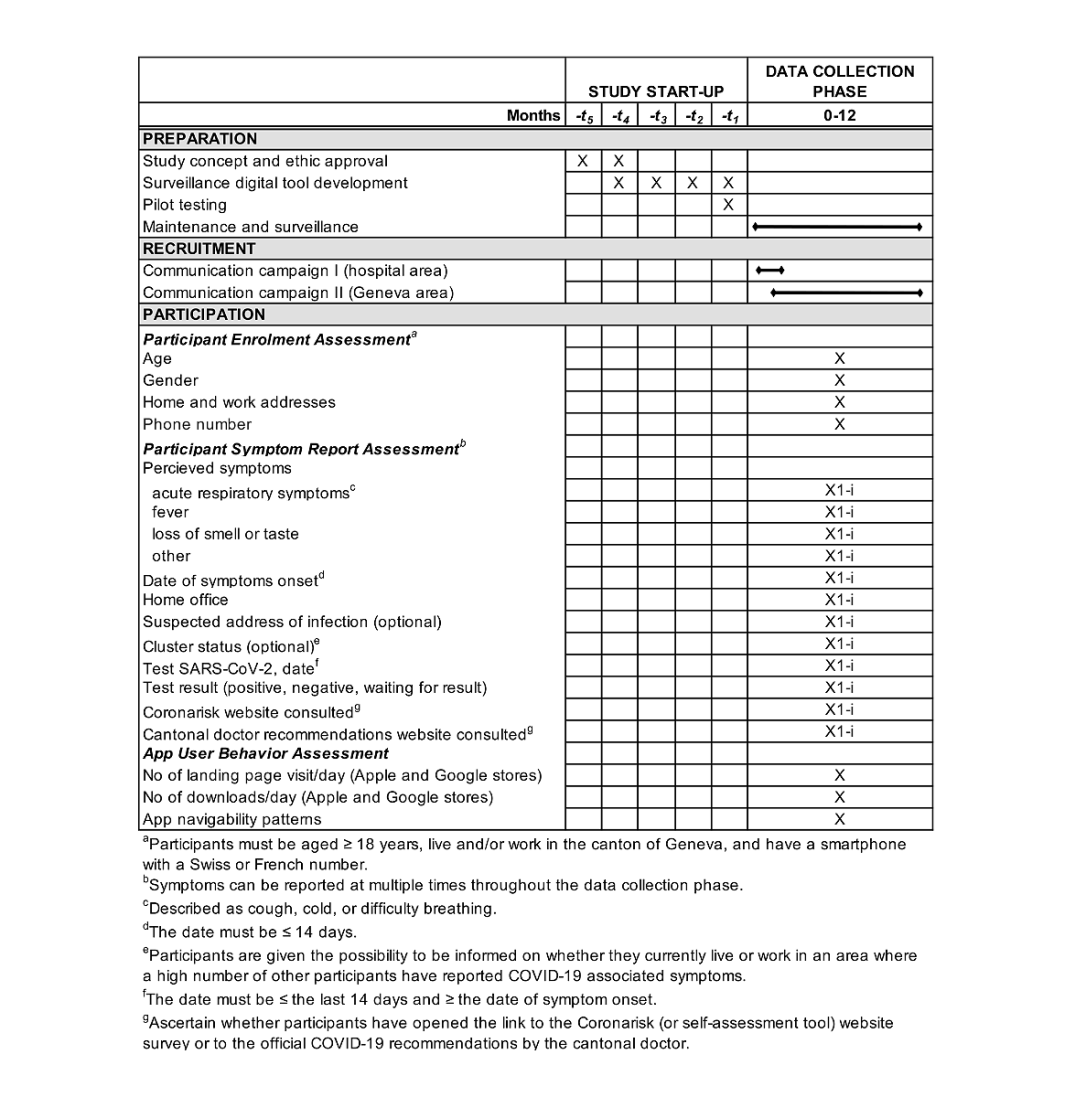
Schedule of study preparation, participant recruitment, and assessments. HUG: Geneva University Hospitals.

Enrollment and follow-up data are collected at study registration and at each report of COVID-19–associated symptoms, respectively (Figure 4). Enrollment data include age (date of birth, age≥18 years), gender (female, male, nonbinary), home and work addresses (street name, street number, postal code vs do not live or work in Geneva), and phone number (Swiss or French prefix number). The data obtained for each symptoms report include perceived symptoms (acute respiratory symptoms [cough, cold, or difficulty breathing], fever, loss of smell or taste, other), date of symptom onset (≤last 14 days), home office (yes vs no), suspected address of infection (street name, street number, postal code vs do not wish to add an additional address), cluster status (I wish vs I do not wish to be notified) if the participant has not yet done the test, SARS-CoV-2 test (yes vs no), date of the test (≤last 14 days and ≥date of symptom onset), and test result (positive, negative, waiting for result). Participants’ cluster status (principal addresses [home and/or work] in vs out) is obtained through the spatiotemporal clustering analyses performed every 24 hours. Lastly, data collection ascertains whether the participant has opened the link to the self-assessment tool website (yes vs no) and the cantonal doctor’s official recommendations (yes vs no). Participants can change their personal addresses at any time under their @choum profile.

Data on adoption, participation, and usage behaviors are collected from the Google Play Store, Apple Store, and CoronApp-HUG app. Data obtained from these sources include the number of CoronApp-HUG landing page visits per day on each store, the number of CoronApp-HUG downloads per day, and app utilization pattern metrics.

### Data Monitoring and Handling

Since the tool’s launch, data are extracted and analyzed weekly by the research team to monitor adoption, participation, and currently active and emerging spatiotemporal clusters. Information on areas currently facing increased COVID-19–associated symptoms may be shared with local public health authorities to guide local interventions. Individual-level data will not be shared at any time, and an assessment of possible participation biases will be conducted before sharing any information to avoid risks of misguiding public health efforts. The evolution of the participation rates and the geographical distribution of participants are used to adapt the communication campaign activities in intensity and location.

All collected data are treated confidentially and securely stored in the HUG-located server, respecting the general data protection regulation legislations of the European Union (GDPR) and Geneva (LIPAD). A unique validation code sent to the participant is used to confirm the participant device’s ownership and reduce abusive system use. The data collected on the frontend of the app are sent to the server at specific time points: upon study registration completion, after being asked about SARS-CoV-2 test results, and every time the validation code is sent (Figure 3). The geocoding (ie, the transformation of addresses into geographic coordinates) is implemented through a completely offline procedure using a reference dataset of Swiss addresses [33]. The geographical masking is conducted before uploading the data to the server, and thus no exact street address or original geographical coordinates are uploaded to the server at any given time. One data manager, responsible for the database maintenance, encrypts the data prior to their extraction for analyses. An opt-out request by the participant results in complete deletion of the account and data.

### Sample Size

The sample size was calculated as ~5000 participants to detect active and emerging spatiotemporal clusters of reported COVID-19–associated symptoms. This analysis assumed a total of 500,000 potential participants aged≥18 years (~400,000 living in and ~100,000 commuting to Geneva for work) [34,35]. Out of this total number, 2% (~10,000) may download the app and half (~5000) are expected to consent to the study. Statistics on health-related mobile app retention rates show that 3% of users are likely to use a health-related app for an entire 30-day use period [36-38]. However, COVID-19 mobile apps have seen better retention rates, with examples such as the UK National Health Service COVID-19 app and the SwissCovid app, which are being used by around 20% of the population [39,40]. A conservative sample size was selected because it is expected to be highly influenced by the evolution of the epidemiological situation.

### Data Analysis

#### Software

Analyses will be conducted using Python 3.8 [41], R 4.0.5 [42], GeoDa 1.18 [43], and QGIS 3.18.1 [44].

#### Prospective Analyses

The prospective detection of spatiotemporal clusters will be achieved using the modified space-time density-based spatial clustering of applications with noise (MST-DBSCAN) algorithm, designed to detect, characterize, and visualize disease cluster evolution [45]. The MST-DBSCAN algorithm is among various density-based clustering methods to detect disease clusters. This modified version of the spatiotemporal DBSCAN has the advantage of considering the transmission relationship between cases, thus incorporating the effect of the incubation period and the ability to detect irregularly shaped clusters [45]. Additionally, density-based methodologies only require symptomatic reports to function, making them particularly suited to the @choum study that collects reports only from users experiencing symptoms. This method determines clusters using a circular spatial scanning window (*EpsS*) and a time window defined by two parameters, *EpsT1* and *EpsT2*, which determine the threshold value for the longest and shortest transmission period, respectively. Similar to the traditional DBSCAN, the MST-DTSCAN algorithm then classifies points into three categories: core, border, and noise points [45,46]. A *core point* is a point with enough spatiotemporal neighbors to be considered the major structure of a cluster with high incidence. The minimum number of neighbors that a point must have to be classified as a core is defined by the parameter *MinPts*. A *border point* is a point that is part of a cluster without being itself the center of a cluster because it does not have enough neighbors. Finally, *noise points* are outliers located in low-incidence areas [45].

To obtain the most up-to-date information on active and emerging clusters, a Python (v3.8) script, based on the package pySDA 0.1.6 [47], is automatically run every 24 hours using the date and masked geographical coordinates (home, work, suspected location of infection) of the symptom reports. This information is used to notify participants reporting COVID-19–associated symptoms of their cluster status (ie, within a cluster or not). The algorithm’s parameters are currently set to *MinPts*=3, *EpsT1*=14, *EpsT2*=1, and *EpsS*=600 meters. The time window is specified according to the current knowledge on incubation and transmission periods [48]. The spatial window of 600 meters represents a good compromise between the resolution of the analysis and privacy preservation [21,25]. The *MinPts* is set to 3 according to the cluster definition used by local health authorities [49]. The parameters used for each daily analysis are stored and adapted according to the latest research on the spatiotemporal scales of COVID-19 transmission. The Python script returns the geographical coordinates of the detected clusters. Only the clusters active at the date of analysis are retained. To avoid geometries in our data, which would require spatial databases, the area of the circular polygon formed by each cluster is approximated using hectometric squares that are uniquely identified and stored using their upper-left coordinates only.

#### Retrospective Analyses

Descriptive statistics of user participation, retention, and collected participant data will be computed using means (SD) and medians (IQR), and frequencies for continuous and categorical variables. These will enable assessing our sample’s representativity compared to the canton of Geneva’s general population.

The number of monthly active users and the number of created and deleted accounts will be monitored during the entire study period, which will enable estimating the retention rate of the @choum study. The performance and the conversion rate of the digital communication campaign will be monitored for each employed social media platform (ie, Twitter, YouTube, Facebook, Instagram, and LinkedIn) by collecting metrics on the number of views and the number of users interacting with each communication post. Additionally, the number of downloads of the CoronApp-HUG and the number of @choum registered users will be monitored weekly following these communication campaigns. Together, this will allow us to estimate the conversion rate of the communication efforts.

We will analyze outbreaks across the entire study period using spatiotemporal and purely spatial statistic methods to understand the evolution of the outbreaks detected over the 12-month data collection phase. Unusual utilization patterns such as repeated reports (ie, >2) of symptoms with positive RT-PCR test results will be classified as “spam” and discarded from retrospective analyses. However, this potential risk of high users falsely inflating reports in location and time has been reduced by limiting users to only report COVID-19–associated systems once every 14 days to ensure that reports correspond to a single episode of COVID-19–associated symptoms. Multiple methods will be used to provide a more complete and robust assessment of the underlying spatiotemporal process [50,51], and include the space-time scan statistics [52], local join count statistics [53], and MST-DBSCAN [45]. The latter will provide a fine-scale characterization of the evolution of clusters in Geneva’s canton across the entire study period. This characterization of clusters into classes (eg, emerge, growth, steady, reduce, merge, move, split) will enable the differentiation of populations and areas of high and low temporal concentration of symptoms (ie, epidemic peakedness) [54]. Lastly, we will examine the association between spatiotemporal cluster characteristics and sociodemographic and environmental determinants of COVID-19–associated symptoms (covariates) using procedures presented in detail elsewhere [22,25].

## Results

The study began on September 1, 2020, and will be completed on February 28, 2022. The @choum study received final approval on November 12, 2020, by the Cantonal Research Ethics Commission of Geneva, Switzerland (2020-01586). A 5-month preparation phase has preceded the 12-month data collection phase starting on September 1, 2020, and February 11, 2021, respectively. Multiple tests performed at various time points throughout the first 4 months of the preparation phase have helped to improve the tool’s user experience. Subsequently, the 1-month pilot phase conducted among 38 pharmacists confirmed that the spatiotemporal clustering analyses worked appropriately, and the feedback helped to improve the user experience and address technical bugs. Since the tool’s launch to the entire population of Geneva on February 11, 2021, and as of May 15, 2021, we have enrolled over 1000 eligible participants. The primary study outcomes are expected to be published in mid-2022.

## Discussion

The @choum study seeks to develop, implement, and test an innovative participatory epidemiological surveillance tool to rapidly detect and prevent clusters of COVID-19–associated symptoms by using precise geographic information. Multiple tests and a 1-month pilot study conducted among 38 pharmacists working in 7 Geneva-based pharmacies have confirmed the proper functioning of the tool. Since the tool’s launch to the entire population of Geneva on February 11, 2021, daily prospective clustering analyses at a local scale help detect active and emerging spatiotemporal clusters, and thus inform citizens and local authorities on areas potentially facing a COVID-19 burden.

The retrospective analyses performed at the end of the study will deliver critical insights into the mechanisms underlying the diffusion dynamics of SARS-CoV-2.

The rationale underlying the @choum study builds on the well-recognized need to rapidly inform key stakeholders (ie, clinical decision-makers, local authorities, and citizens) about geographical areas facing a higher burden of disease [6,9,54]. Prioritizing areas and populations of high COVID-19 burden has been critical since the start of the SARS-CoV-2 pandemic and will continue during the following months as the vaccination campaign expands. For example, the province of Ontario, Canada, has recently expanded vaccination eligibility criteria to all adults living in hotspot neighborhoods [55]. Similarly, our group has shown that clusters observed among a sample of over 3000 confirmed COVID-19 cases were more persistent in the socioeconomically disadvantaged Geneva-area neighborhoods, which could be targeted in priority for intervention [25]. However, most existing participatory syndromic surveillance initiatives are not designed to collect high-resolution spatiotemporal data [56]. The @choum study tackles this challenge by using modern spatiotemporal methods and precise location data while protecting the user’s privacy, enabling potentially more efficient and effective management of the SARS-CoV-2 pandemic. A few notable studies have already taken a similar approach using fine-scale geographic information in combination with spatiotemporal clustering analyses [7,16]. For example, Leal-Neto et al [7] used a combination of data from traditional and participatory surveillance to detect spatial clusters; however, the temporal component was not considered due to the potential latency of 14 days on COVID-19 cases. The MST-DBSCAN algorithm employed in the @choum study tackles this challenge by incorporating the incubation period effect.

Since the start of the pandemic, a growing body of literature has highlighted the existence of health inequalities in COVID-19 infection and mortality [57-59]. However, most studies have been performed in the United States and United Kingdom. In Switzerland, a study published by our group in early 2021 revealed important sociodemographic determinants of epidemic spread at the local level [25]. Findings from the @choum study will provide critical insights into the mechanisms underlying the diffusion dynamics of SARS-CoV-2 by performing retrospective analyses of collected data, and exploring the associations between cluster characteristics and sociodemographic and environmental determinants. Indeed, the shape of COVID-19 epidemics is an area of great scientific interest [60,61], and the use of spatiotemporal clustering may offer an innovative tool to better understand the determinants of peaked or prolonged epidemics at a fine geographic scale.

A distinctive feature of the @choum tool lies in the unique possibility for participants who have not yet been tested to be informed on their current cluster status (ie, whether they currently live or work in an area where a high number of other participants have reported COVID-19–associated symptoms). In the context of testing fatigue [5], this returned information on the participants’ cluster status could promote testing among symptomatic participants and reduce the delay of notification inherent to traditional epidemiological surveillance systems [8,9]. Moreover, because @choum is integrated into an app for COVID-19 resources and information, and is linked to other official COVID-19 recommendation websites, it supports the centralization of local COVID-19 communications [6]. These may be crucial, as getting mixed messages from various sources can reduce compliance with preventive measures [62,63].

Several limitations of this study merit comment. First, generalization will be limited to the adult population living or working in the canton of Geneva. Second, adoption and utilization of the @choum tool might suffer from a participation bias, as attitudes toward digital COVID-19 public health tools such as contact tracing apps vary among sociodemographic groups [64,65]. However, we developed and implemented several strategies to foster participation across sociodemographic groups and ensure user retention. We implemented a comprehensive communication campaign using numerous online and offline channels. In implementing this campaign, potential competition between the @choum study and the preexisting contact tracing app (SwissCovid) [32] has been considered; @choum was explicitly designed to avoid this possibility, notably by encouraging the adoption of SwissCovid and explaining their complementarity. We also aimed to allow users to rapidly and easily report symptoms, which could improve participation and retention rates compared to other participatory syndromic surveillance systems requiring frequent use or periodical inputs. Third, geographic data are limited to fixed coordinates, and thus occupations involving high mobility (eg, in-home nurses, taxi drivers) are not represented.

To the best of our knowledge, @choum is the first participatory epidemiological surveillance system combining fine-scale spatiotemporal data with prospective clustering analyses. Such a tool could be helpful to effectively prevent the spread of COVID-19 and related diseases. This tool has the potential to provide timely and precise geographical information to encourage infection control interventions at a local scale and public health recommendations compliance. The study findings will also advance other research contributions investigating the complex relationship of environment and health using novel digital technologies. It is thus hoped that the @choum study will be a valuable support in the fight against COVID-19.

## Appendix

Multimedia Appendix 1 All screens related to study participation.

## Abbreviations

eIC: electronic informed consent
EPFL: Swiss Federal Institute of Technology Lausanne
HUG: Geneva University Hospitals
MST-DBSCAN: modified space-time density-based spatial clustering of applications with noise
RT-PCR: real-time polymerase chain reaction
SFOPH: Swiss Federal Office of Public Health
UNIGE: University of Geneva
UP: University of Paris
