# Supplementary material for: Detection of Spatiotemporal Clusters of COVID-19–Associated Symptoms and Prevention Using a Participatory Surveillance App: Protocol for the @choum Study
Source: JMIR Res Protoc. 2021 Oct 6;10(10):e30444. doi: 10.2196/30444 (PMC8496683; doi:10.2196/30444)

Multimedia Appendix of Supplementary Files

Appendix 1. All screens related to study participation

(A) Consent & registration

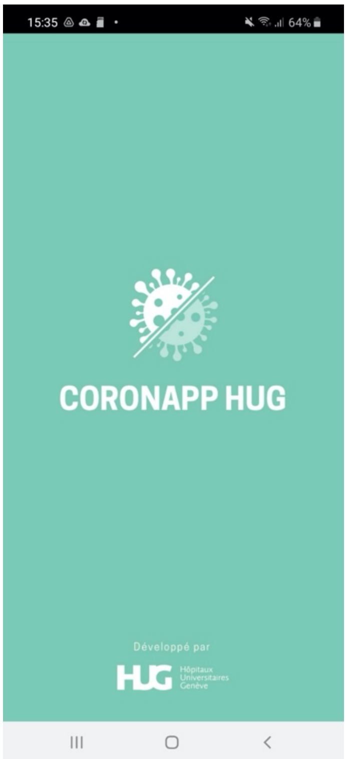

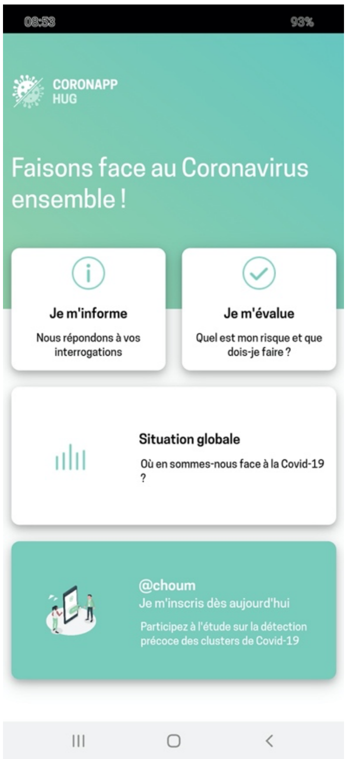

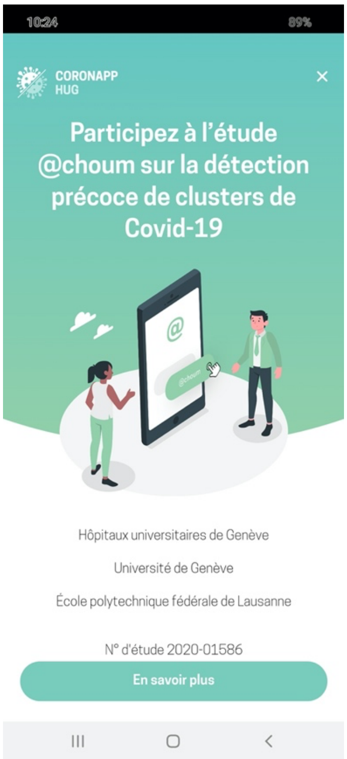

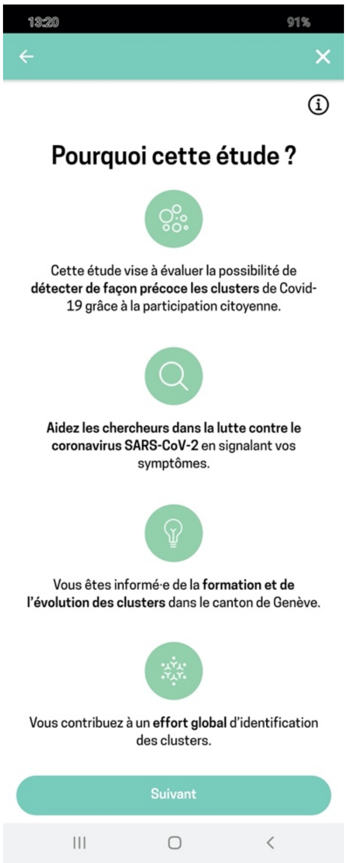

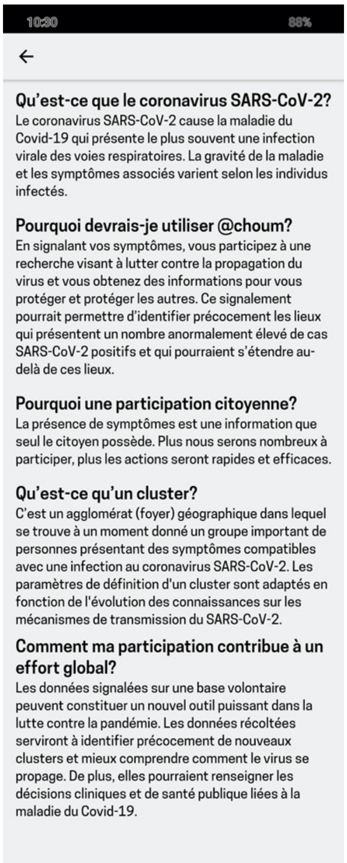

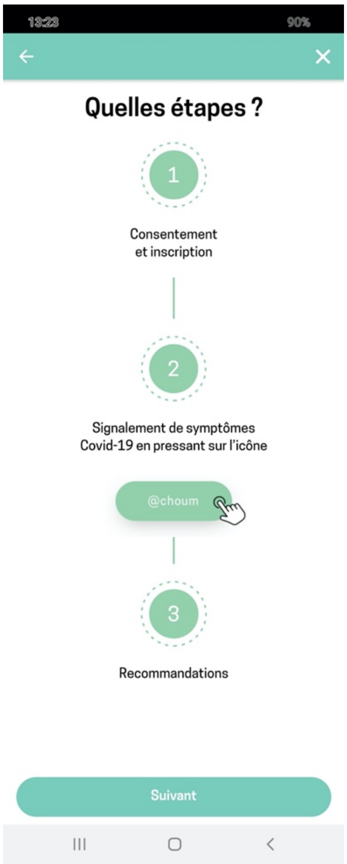

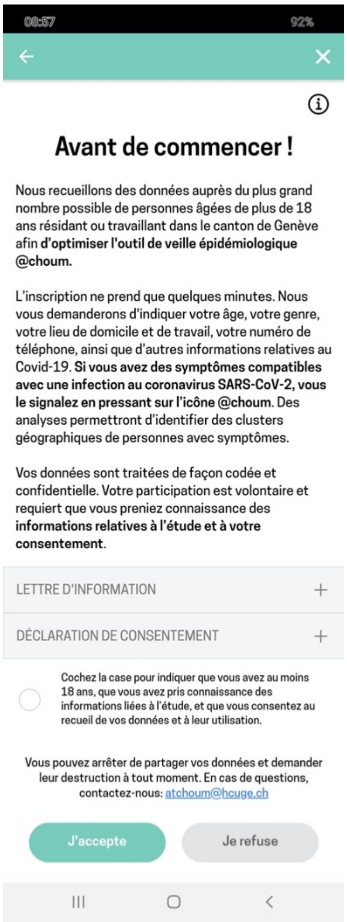
08:57
92%
Avant de commencer !
Nous recueillons des données auprès du plus grand nombre possible de personnes âgées de plus de 18 ans résidant ou travaillant dans le canton de Genève afin d'optimiser l'outil de veille épidémiologique @choum.
L'inscription ne prend que quelques minutes. Nous vous demanderons d'indiquer votre âge, votre genre, votre lieu de domicile et de travail, votre numéro de téléphone, ainsi que d'autres informations relatives au Covid-19. Si vous avez des symptômes compatibles avec une infection au coronavirus SARS-CoV-2, vous le signalez en pressant sur l'icône @choum. Des analyses permettront d'identifier des clusters géographiques de personnes avec symptômes.
Vos données sont traitées de façon codée et confidentielle. Votre participation est volontaire et requiert que vous preniez connaissance des informations relatives à l'étude et à votre consentement.
LETTRE D'INFORMATION
DÉCLARATION DE CONSENTEMENT
Cochez la case pour indiquer que vous avez au moins 18 ans, que vous avez pris connaissance des informations liées à l'étude, et que vous consentez au recueil de vos données et à leur utilisation.
Vous pouvez arrêter de partager vos données et demander leur destruction à tout moment. En cas de questions, contactez-nous: atchoum@hcuge.ch
J'accepte
Je refuse

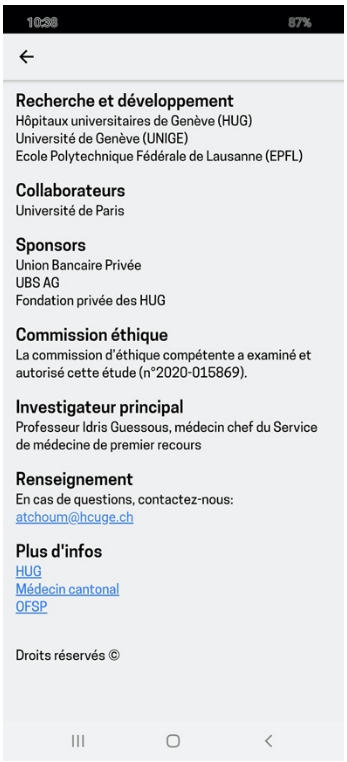
10:38
87%
Recherche et développement
Hôpitaux universitaires de Genève (HUG)
Université de Genève (UNIGE)
Ecole Polytechnique Fédérale de Lausanne (EPFL)
Collaborateurs
Université de Paris
Sponsors
Union Bancaire Privée
UBS AG
Fondation privée des HUG
Commission éthique
La commission d'éthique compétente a examiné et autorisé cette étude (n°2020-015869).
Investigateur principal
Professeur Idris Guessous, médecin chef du Service de médecine de premier recours
Renseignement
En cas de questions, contactez-nous:
atchoum@hcuge.ch
Plus d'infos
HUG
Médecin cantonal
OFSP
Droits réservés ©

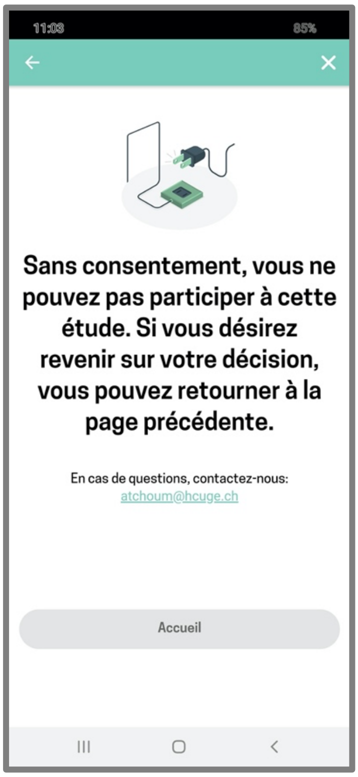
11:03
85%
Sans consentement, vous ne pouvez pas participer à cette étude. Si vous désirez revenir sur votre décision, vous pouvez retourner à la page précédente.
En cas de questions, contactez-nous:
atchoum@hcuge.ch
Accueil

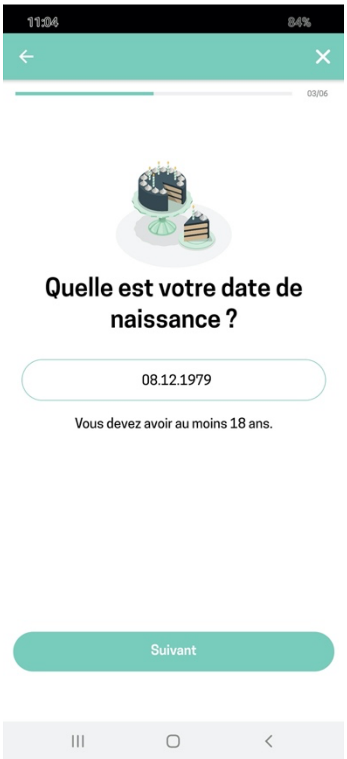
11:04
84%
03/06
Quelle est votre date de naissance ?
08.12.1979
Vous devez avoir au moins 18 ans.
Suivant

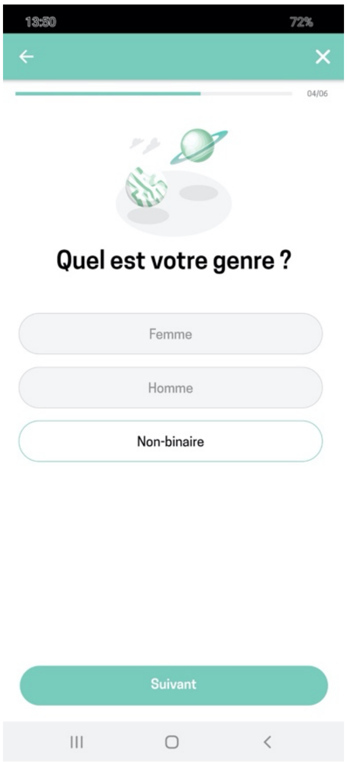
13:50
72%
04/06
Quel est votre genre ?
Femme
Homme
Non-binaire
Suivant

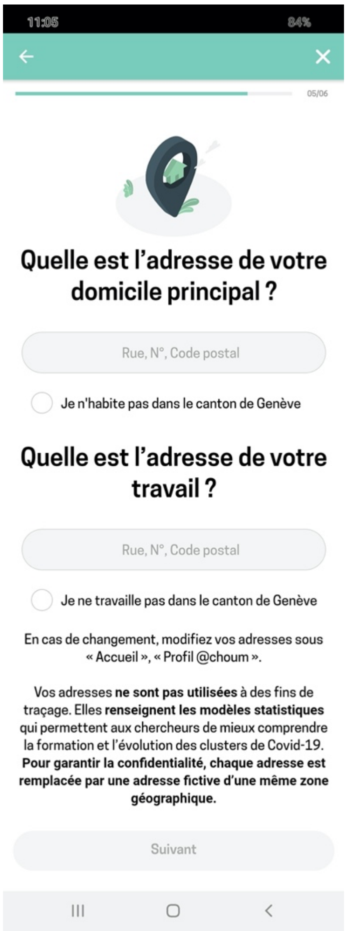
11:05
84%
05/06
Quelle est l'adresse de votre domicile principal ?
Rue, N°, Code postal
Je n'habite pas dans le canton de Genève
Quelle est l'adresse de votre travail ?
Rue, N°, Code postal
Je ne travaille pas dans le canton de Genève
En cas de changement, modifiez vos adresses sous « Accueil », « Profil @choum ».
Vos adresses ne sont pas utilisées à des fins de traçage. Elles renseignent les modèles statistiques qui permettent aux chercheurs de mieux comprendre la formation et l'évolution des clusters de Covid-19. Pour garantir la confidentialité, chaque adresse est remplacée par une adresse fictive d'une même zone géographique.
Suivant

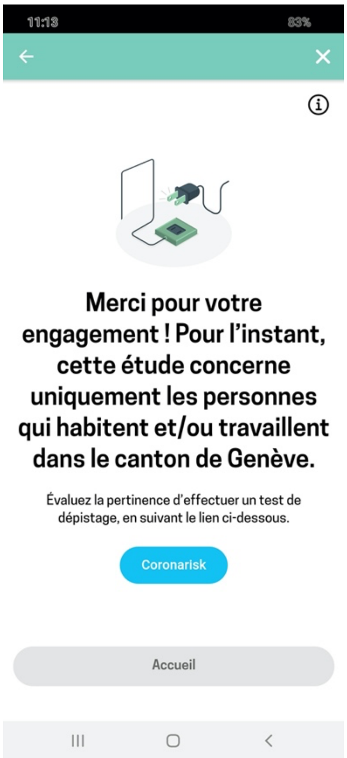
11:13
83%
Merci pour votre engagement ! Pour l'instant, cette étude concerne uniquement les personnes qui habitent et/ou travaillent dans le canton de Genève.
Évaluez la pertinence d'effectuer un test de dépistage, en suivant le lien ci-dessous.
Coronarisk
Accueil

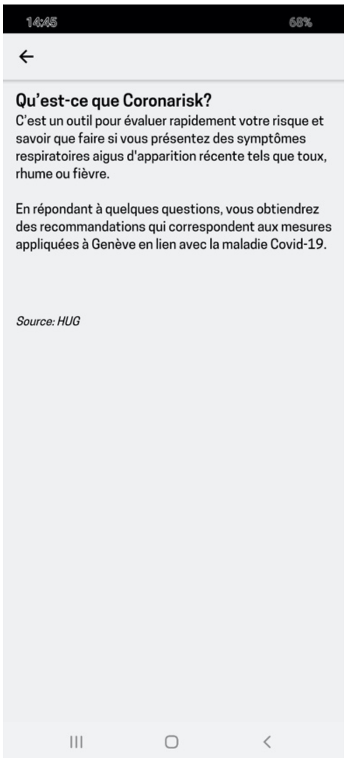
14:45
68%
Qu'est-ce que Coronarisk?
C'est un outil pour évaluer rapidement votre risque et savoir que faire si vous présentez des symptômes respiratoires aigus d'apparition récente tels que toux, rhume ou fièvre.
En répondant à quelques questions, vous obtiendrez des recommandations qui correspondent aux mesures appliquées à Genève en lien avec la maladie Covid-19.
Source: HUG

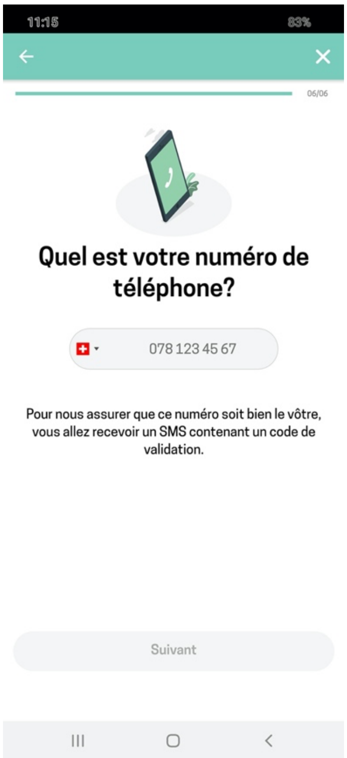
11:15
83%
06/06
Quel est votre numéro de téléphone?
078 123 45 67
Pour nous assurer que ce numéro soit bien le vôtre, vous allez recevoir un SMS contenant un code de validation.
Suivant

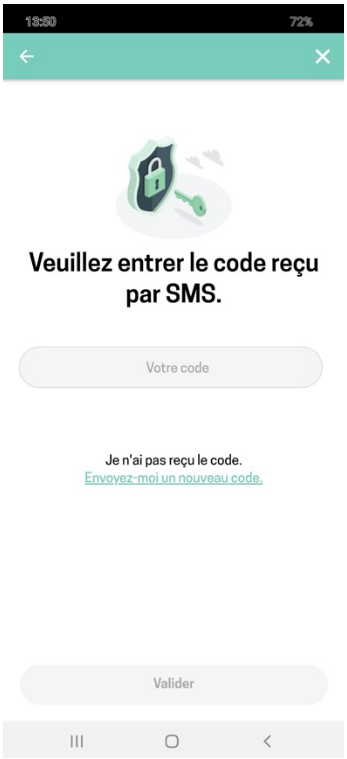
13:50
72%
Veuillez entrer le code reçu par SMS.
Votre code
Je n'ai pas reçu le code.
Envoyez-moi un nouveau code.
Valider

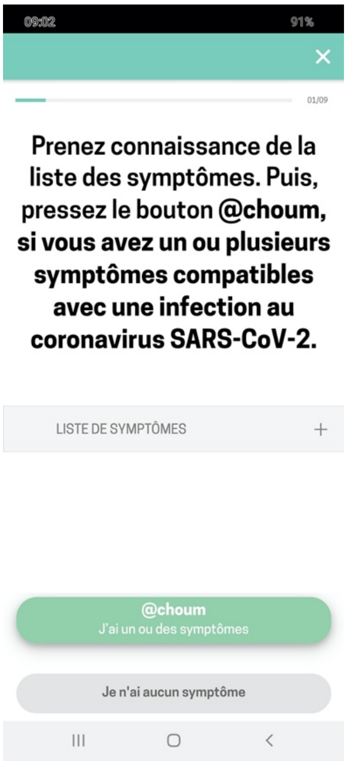
09:02
91%
01/09
Prenez connaissance de la liste des symptômes. Puis, pressez le bouton @choum, si vous avez un ou plusieurs symptômes compatibles avec une infection au coronavirus SARS-CoV-2.
LISTE DE SYMPTÔMES
@choum
J'ai un ou des symptômes
Je n'ai aucun symptôme

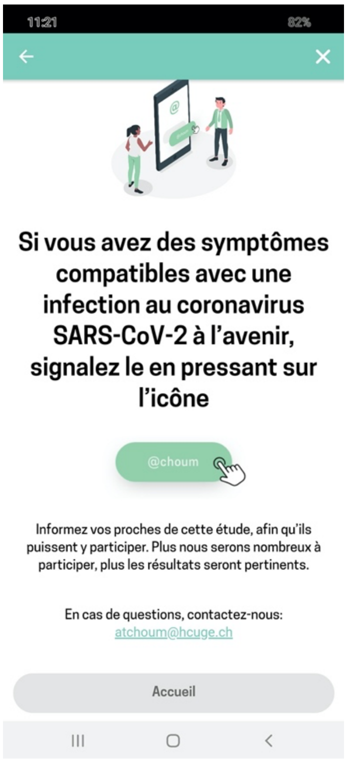
11:21
82%
Si vous avez des symptômes compatibles avec une infection au coronavirus SARS-CoV-2 à l'avenir, signalez le en pressant sur l'icône
@choum
Informez vos proches de cette étude, afin qu'ils puissent y participer. Plus nous serons nombreux à participer, plus les résultats seront pertinents.
En cas de questions, contactez-nous:
atchoum@hcuge.ch
Accueil

(B) Symptom report(s)

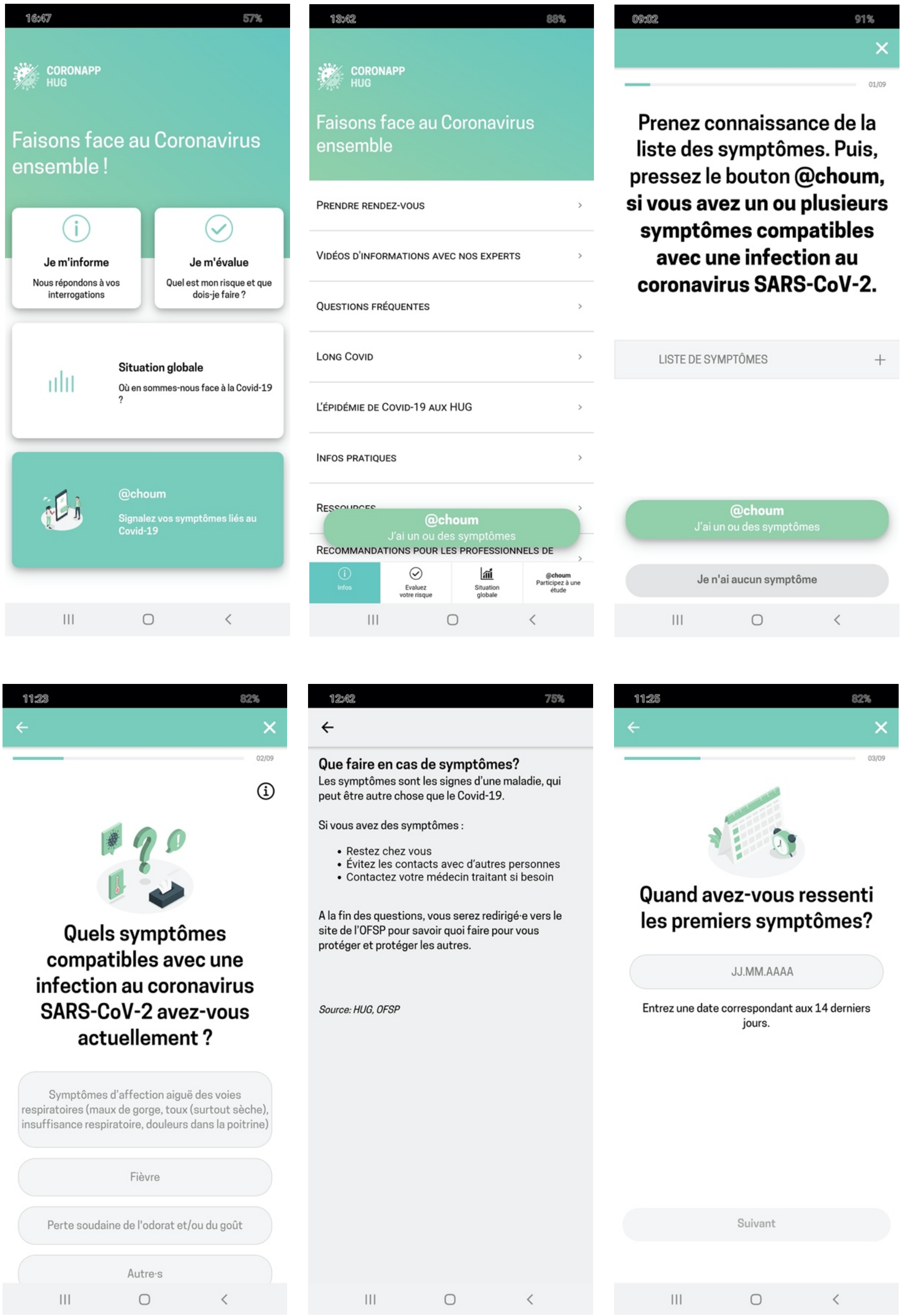

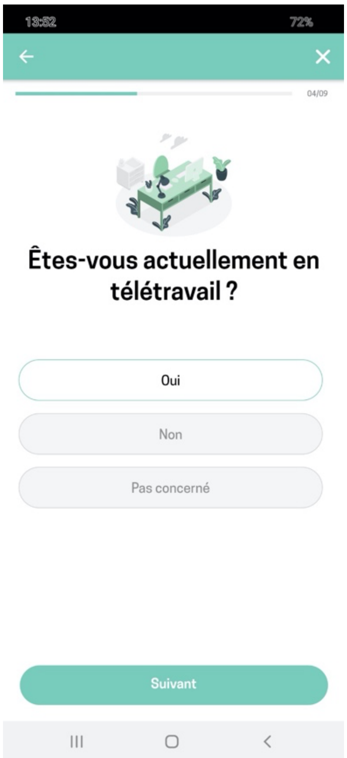
13:32
72%
04/09
Êtes-vous actuellement en télétravail ?
Oui
Non
Pas concerné
Suivant

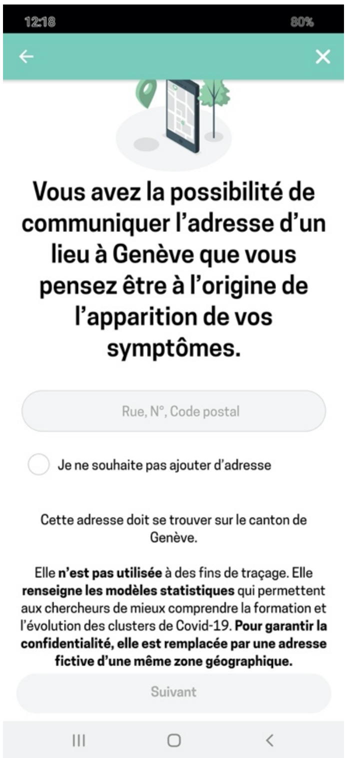
12:18
80%
Vous avez la possibilité de communiquer l'adresse d'un lieu à Genève que vous pensez être à l'origine de l'apparition de vos symptômes.
Rue, N°, Code postal
Je ne souhaite pas ajouter d'adresse
Cette adresse doit se trouver sur le canton de Genève.
Elle **n'est pas utilisée** à des fins de traçage. Elle **renseigne les modèles statistiques** qui permettent aux chercheurs de mieux comprendre la formation et l'évolution des clusters de Covid-19. **Pour garantir la confidentialité, elle est remplacée par une adresse fictive d'une même zone géographique.**
Suivant

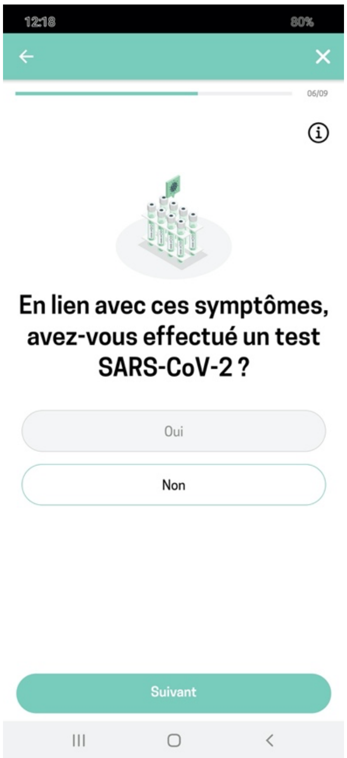
12:18
80%
06/09
En lien avec ces symptômes, avez-vous effectué un test SARS-CoV-2 ?
Oui
Non
Suivant

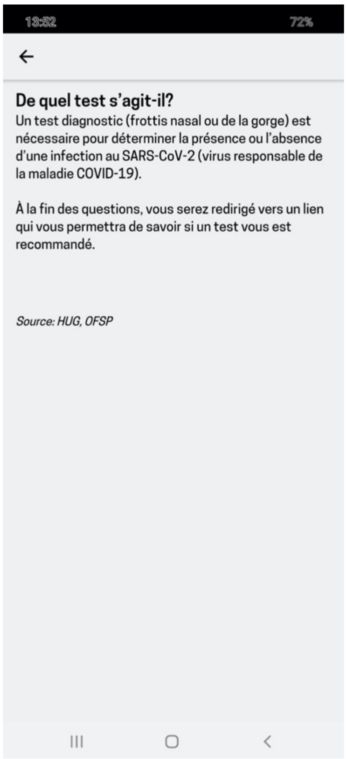
13:32
72%
De quel test s'agit-il?
Un test diagnostic (frottis nasal ou de la gorge) est nécessaire pour déterminer la présence ou l'absence d'une infection au SARS-CoV-2 (virus responsable de la maladie COVID-19).
À la fin des questions, vous serez redirigé vers un lien qui vous permettra de savoir si un test vous est recommandé.
Source: HUG, OFSP

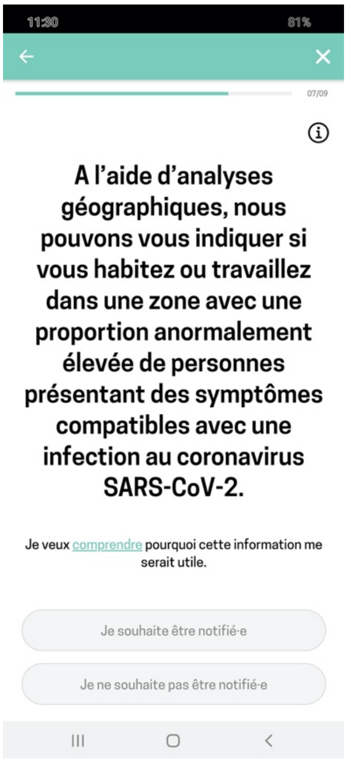
11:30
81%
07/09
A l'aide d'analyses géographiques, nous pouvons vous indiquer si vous habitez ou travaillez dans une zone avec une proportion anormalement élevée de personnes présentant des symptômes compatibles avec une infection au coronavirus SARS-CoV-2.
Je veux comprendre pourquoi cette information me serait utile.
Je souhaite être notifié·e
Je ne souhaite pas être notifié·e

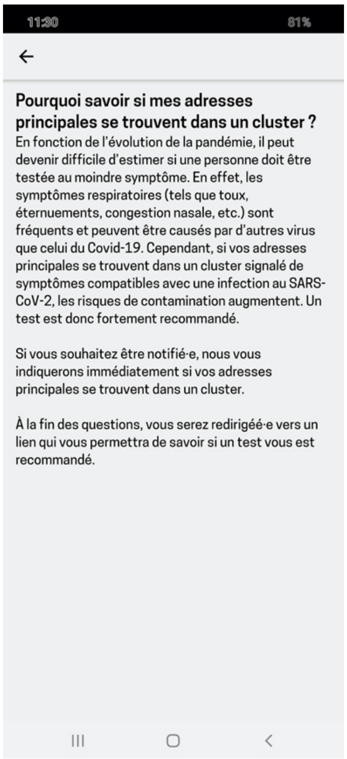
11:30
81%
Pourquoi savoir si mes adresses principales se trouvent dans un cluster ?
En fonction de l'évolution de la pandémie, il peut devenir difficile d'estimer si une personne doit être testée au moindre symptôme. En effet, les symptômes respiratoires (tels que toux, éternuements, congestion nasale, etc.) sont fréquents et peuvent être causés par d'autres virus que celui du Covid-19. Cependant, si vos adresses principales se trouvent dans un cluster signalé de symptômes compatibles avec une infection au SARS-CoV-2, les risques de contamination augmentent. Un test est donc fortement recommandé.
Si vous souhaitez être notifié·e, nous vous indiquerons immédiatement si vos adresses principales se trouvent dans un cluster.
À la fin des questions, vous serez redirigéé·e vers un lien qui vous permettra de savoir si un test vous est recommandé.

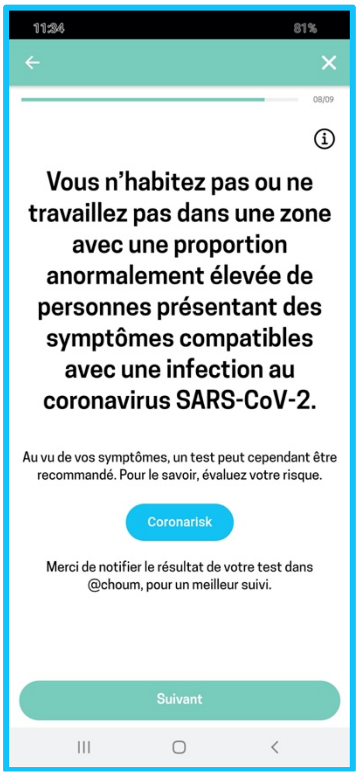
11:34
81%
08/09
Vous n'habitez pas ou ne travaillez pas dans une zone avec une proportion anormalement élevée de personnes présentant des symptômes compatibles avec une infection au coronavirus SARS-CoV-2.
Au vu de vos symptômes, un test peut cependant être recommandé. Pour le savoir, évaluez votre risque.
Coronarisk
Merci de notifier le résultat de votre test dans @choum, pour un meilleur suivi.
Suivant

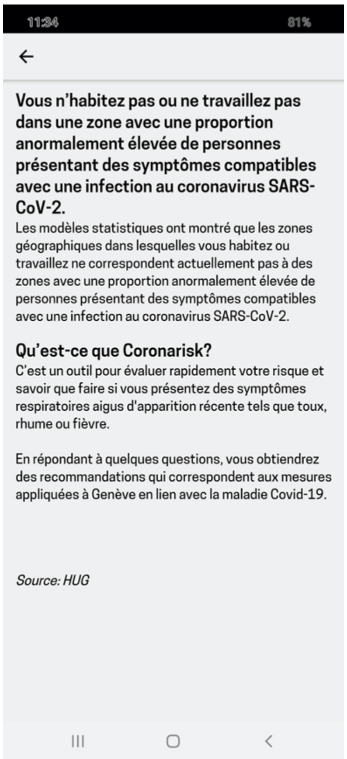
11:34
81%
Vous n'habitez pas ou ne travaillez pas dans une zone avec une proportion anormalement élevée de personnes présentant des symptômes compatibles avec une infection au coronavirus SARS-CoV-2.
Les modèles statistiques ont montré que les zones géographiques dans lesquelles vous habitez ou travaillez ne correspondent actuellement pas à des zones avec une proportion anormalement élevée de personnes présentant des symptômes compatibles avec une infection au coronavirus SARS-CoV-2.
Qu'est-ce que Coronarisk?
C'est un outil pour évaluer rapidement votre risque et savoir que faire si vous présentez des symptômes respiratoires aigus d'apparition récente tels que toux, rhume ou fièvre.
En répondant à quelques questions, vous obtiendrez des recommandations qui correspondent aux mesures appliquées à Genève en lien avec la maladie Covid-19.
Source: HUG

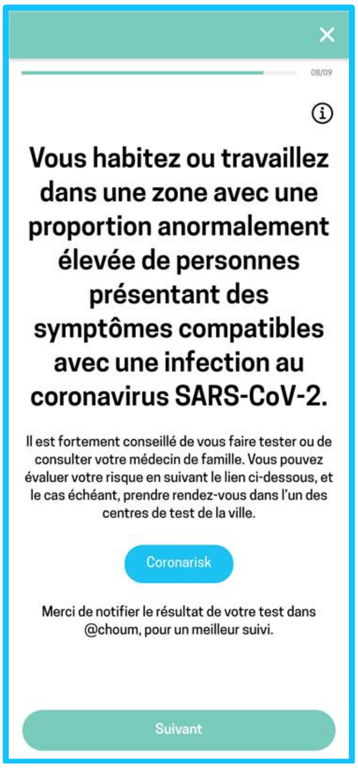
08/09
Vous habitez ou travaillez dans une zone avec une proportion anormalement élevée de personnes présentant des symptômes compatibles avec une infection au coronavirus SARS-CoV-2.
Il est fortement conseillé de vous faire tester ou de consulter votre médecin de famille. Vous pouvez évaluer votre risque en suivant le lien ci-dessous, et le cas échéant, prendre rendez-vous dans l'un des centres de test de la ville.
Coronarisk
Merci de notifier le résultat de votre test dans @choum, pour un meilleur suivi.
Suivant

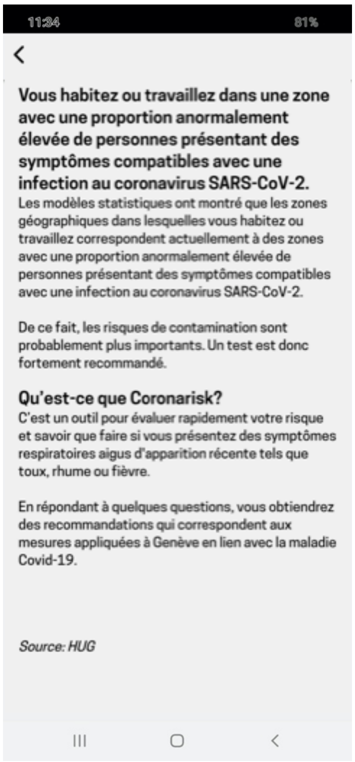
11:34
81%
Vous habitez ou travaillez dans une zone avec une proportion anormalement élevée de personnes présentant des symptômes compatibles avec une infection au coronavirus SARS-CoV-2.
Les modèles statistiques ont montré que les zones géographiques dans lesquelles vous habitez ou travaillez correspondent actuellement à des zones avec une proportion anormalement élevée de personnes présentant des symptômes compatibles avec une infection au coronavirus SARS-CoV-2.
De ce fait, les risques de contamination sont probablement plus importants. Un test est donc fortement recommandé.
Qu'est-ce que Coronarisk?
C'est un outil pour évaluer rapidement votre risque et savoir que faire si vous présentez des symptômes respiratoires aigus d'apparition récente tels que toux, rhume ou fièvre.
En répondant à quelques questions, vous obtiendrez des recommandations qui correspondent aux mesures appliquées à Genève en lien avec la maladie Covid-19.
Source: HUG

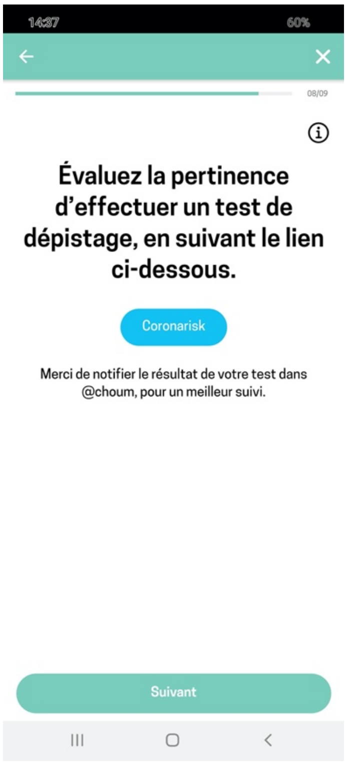
14:37
60%
08/09
Évaluez la pertinence d'effectuer un test de dépistage, en suivant le lien ci-dessous.
Coronarisk
Merci de notifier le résultat de votre test dans @choum, pour un meilleur suivi.
Suivant

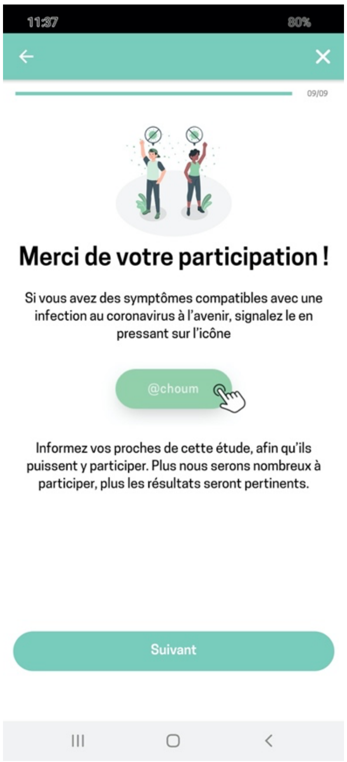
11:37
80%
09/09
Merci de votre participation !
Si vous avez des symptômes compatibles avec une infection au coronavirus à l'avenir, signalez le en pressant sur l'icône
@choum
Informez vos proches de cette étude, afin qu'ils puissent y participer. Plus nous serons nombreux à participer, plus les résultats seront pertinents.
Suivant

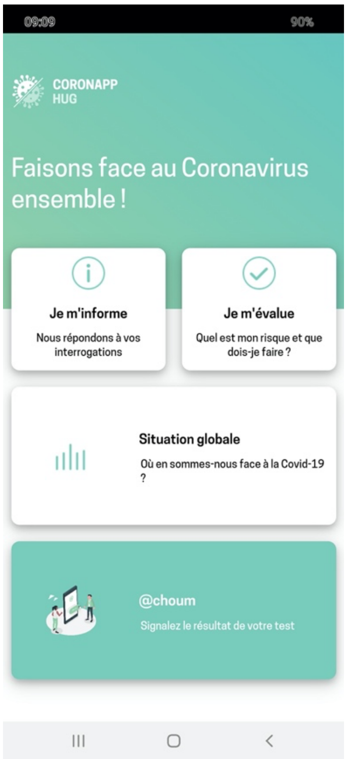
09:09
90%
CORONAPP
HUG
Faisons face au Coronavirus ensemble !
Je m'informe
Nous répondons à vos interrogations
Je m'évalue
Quel est mon risque et que dois-je faire ?
Situation globale
Où en sommes-nous face à la Covid-19 ?
@choum
Signalez le résultat de votre test

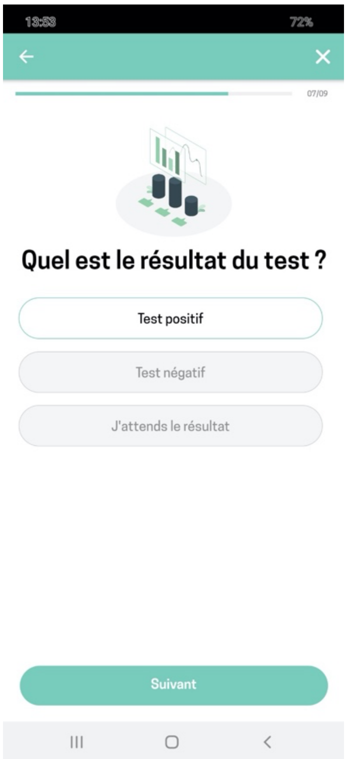
13:53
72%
07/09
Quel est le résultat du test ?
Test positif
Test négatif
J'attends le résultat
Suivant

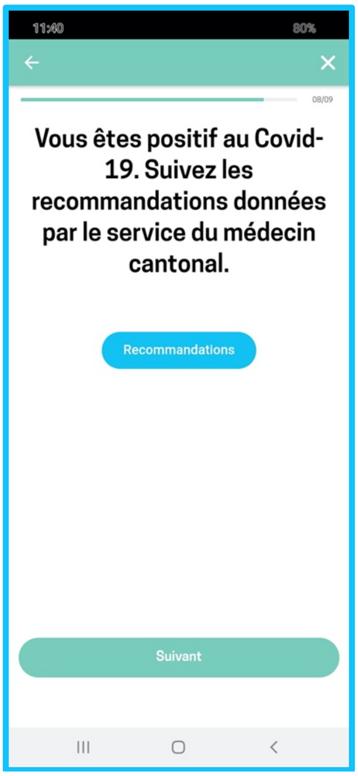
11:40
80%
08/09
Vous êtes positif au Covid-19. Suivez les recommandations données par le service du médecin cantonal.
Recommandations
Suivant

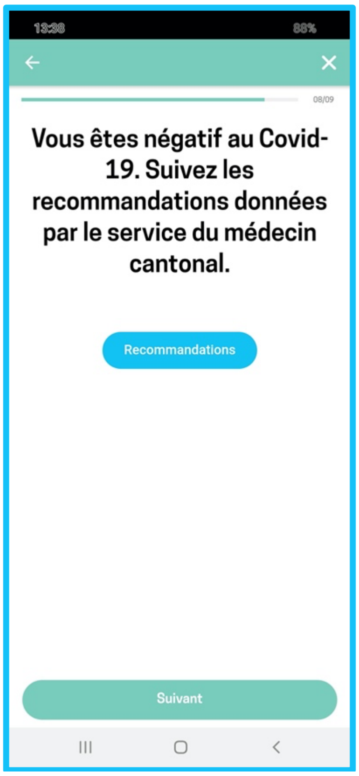
13:38
88%
08/09
Vous êtes négatif au Covid-19. Suivez les recommandations données par le service du médecin cantonal.
Recommandations
Suivant

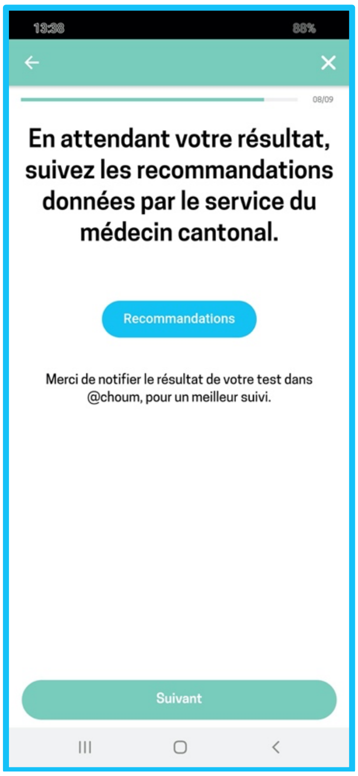
13:38
88%
08/09
En attendant votre résultat, suivez les recommandations données par le service du médecin cantonal.
Recommandations
Merci de notifier le résultat de votre test dans @choum, pour un meilleur suivi.
Suivant

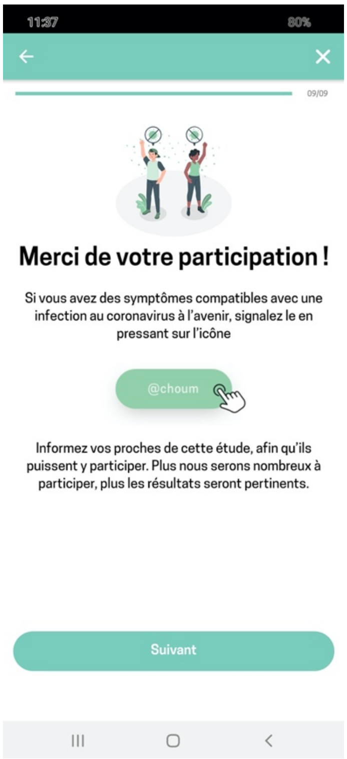
11:37
80%
09/09
Merci de votre participation !
Si vous avez des symptômes compatibles avec une infection au coronavirus à l'avenir, signalez le en pressant sur l'icône
@choum
Informez vos proches de cette étude, afin qu'ils puissent y participer. Plus nous serons nombreux à participer, plus les résultats seront pertinents.
Suivant

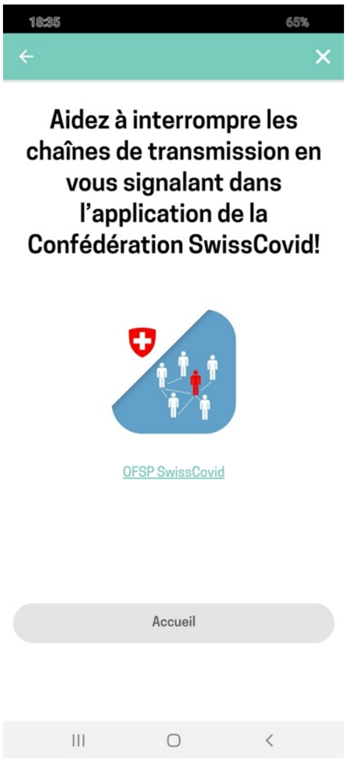

Account & data suppression

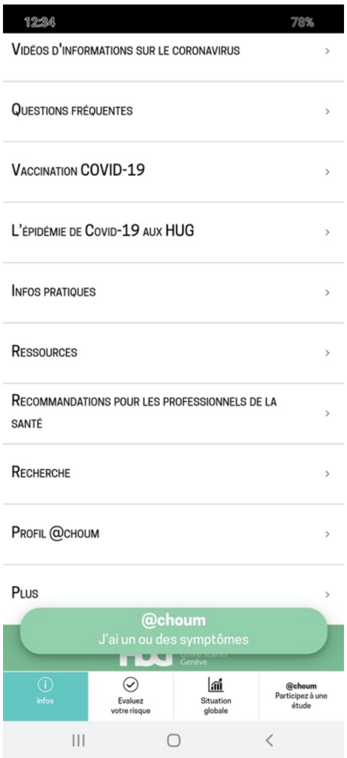

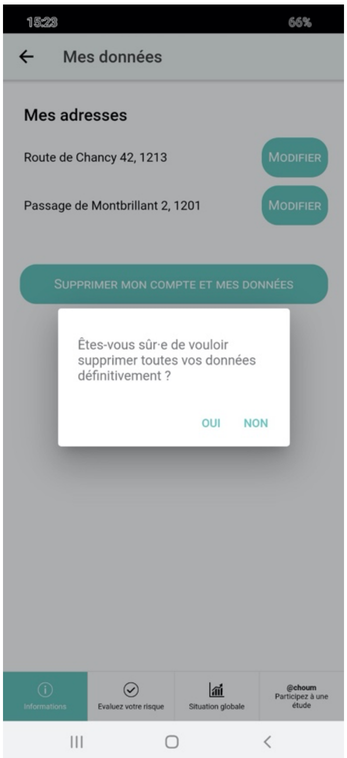

Supplement: Multimedia Appendix 1 [file resprot_v10i10e30444_app1.pdf]
